# Tied Houses in Surgical Practice

**Published:** 1920-05-22

**Authors:** 


### THE EDITOR'S LETTER-BOX.

**Correspondence on all subjects is invited, but we cannot in any way be responsible for the opinions expressed by our correspondents, who must give their name and address as a guarantee of good faith, but not necessarily for publication. Correspondents are reminded that brevity of style and conciseness of statement greatly facilitate early insertion.**

### Tied Houses in Surgical Practice.

*To the Editor of* The Hospital.

Sir,—I have been much interested in the letters which have appeared in your columns from consultants relating to dichotomy. The practice is condemned without reserve by all the writers. But I regret that no remedial or preventive measures have been suggested by them; the nearest approach to these being the comment of Sir George Makins that fee-splitting is definitely forbidden to Fellows of the American College of Surgeons, but even Sir George Makins does not appear to suggest that such a recognised prohibition should be imposed upon the surgeons of this country.

The majority of your correspondents are content with such general expressions of opinion as that "the service of the public and the good of the patient must be the essential considerations," "I had hoped that the whisperings of such traffic had faded away," "I have always felt that the system . . . was most objectionable," "I do not approve of touting in any form," and so forth. We might make somewhat similar remarks upon murder and other crimes, but it is hardly to be supposed that mere expressions of opinion, however lofty, would deter a malefactor from his contemplated purposes.

My original article was inspired by a particular instance which had come to my knowledge, and I mentioned the main details. In this instance the surgeon's position was one of great difficulty, and I wish that some suggestions of a practical nature bearing upon the prevention of dichotomy in such a case had been forthcoming.

As regards the responsibility of the general practitioner in recommending a particular surgeon, we know quite well that factors other than the surgeon's experience and skill enter into the matter of selection. What about the general practitioners who "do their own surgery," and who, according to the medical correspondent of a leading daily paper, "do it remarkably well"? Are these men really as competent as the individual who devotes himself entirely to surgery, or are they, in undertaking surgical procedures themselves, influenced in any way by financial considerations?

Now I have two suggestions to make. The first of these is that the views of others than consultants should be obtained upon the subject of dichotomy and the kindred one of surgical operations performed by general practitioners. The second one is that those in authority should insist upon the taking of an oath, based upon Hippocratic lines, by all members of the profession who undertake to perform surgical operations upon the public.

I am, yours truly,

The Writer of the Original Article.

